# Clinical Evaluation of Accuracy and Speed Across Three Intraoral Scanners

**DOI:** 10.3390/medicina62040622

**Published:** 2026-03-25

**Authors:** Andrei-Bogdan Faur, Anca-Elena Anghel-Lorinți, Anca Jivănescu

**Affiliations:** 1 TADERP Research Center, Department of Prosthodontics, University of Medicine and Pharmacy “Victor Babes”, B-dul Revolutiei 1989, No. 9, 300580 Timisoara, Romania; jivanescu.anca@umft.ro; 2 Department of Prosthodontics, University of Medicine and Pharmacy “Victor Babes”, B-dul Revolutiei 1989, No. 9, 300580 Timisoara, Romania; anca.anghel@umft.ro

**Keywords:** intraoral scanners, digital dental impressions, accuracy (trueness and precision)

## Abstract

*Background and Objectives*: To evaluate and compare the accuracy (trueness and precision) and scanning speed of three intraoral scanners: Medit i700, Primescan 1, and COXO DL-300P, under standardized clinical conditions, using a digitized high-precision analog impression as the reference. *Materials and Methods*: A patient requiring fixed prosthetic treatment on natural abutments (2.5, 2.7, 3.5, 3.8) was selected. Ten sequential full-arch scans were performed with each scanner in rotating order. Scanning time was recorded for each full-arch acquisition. Accuracy analysis focused on regions of interest: the upper jaw included abutments with adjacent mucosa, the lower jaw included only abutment surfaces. A VPS impression was taken, poured in type IV stone, and digitized with a high-accuracy desktop scanner to serve as the reference. All datasets were analyzed in Geomagic Control X, and statistics were performed using MedCalc (α = 0.05). *Results*: Upper Jaw (Abutments and Mucosa): The Medit i700 achieved the highest trueness (100.3 ± 6.6 µm), outperforming Primescan and COXO (*p* = 0.008). COXO showed the best precision, while Primescan demonstrated the greatest variability (*p* < 0.0001). Primescan produced the fastest scans (72.5 ± 3.8 s) (*p* = 0.001). Lower Jaw: (Abutment Surface Only): Medit yielded superior trueness (193.1 ± 63.4 µm) compared with Primescan and COXO (*p* = 0.005). Precision varied significantly among devices, with COXO presenting the greatest inconsistency (*p* = 0.0004). Scan times did not differ significantly (*p* = 0.068). *Conclusions*: Medit i700 demonstrated the highest trueness, Primescan delivered the fastest scans but variable results, and COXO showed acceptable but inconsistent precision.

## 1. Introduction

Digital intraoral scanners (IOS) have transformed prosthodontic workflows by replacing conventional impressions with faster, patient-friendly, and digitally integrated alternatives. Their adoption has led to greater clinical efficiency and improved communication with dental laboratories [1,2]. However, the success of digital impressions depends critically on the accuracy of the scans, especially in complex cases involving multiple abutments or partially edentulous regions [3].

Intraoral scanners rely on different optical acquisition technologies to capture three-dimensional dental structures, including structured light projection, confocal microscopy, triangulation, and active wavefront sampling. These technologies differ in their ability to capture surface details, manage reflective or moist environments, and maintain tracking stability during longer scans. Confocal-based systems can acquire depth information by focusing on different distances, whereas structured light systems rely on projecting patterns onto the scanned surface and reconstructing geometry from image distortion. Each approach presents advantages and limitations that may influence scanning accuracy, particularly in extended scans involving soft tissues or edentulous regions [1,4].

In prosthodontics, accuracy comprises two components: trueness, the closeness of a measurement to the actual geometry of the scanned object, and precision, the consistency or repeatability of repeated measurements [5]. Literature indicates that an internal and marginal fit between 50 and 120 µm is generally considered clinically acceptable, though several authors propose 50–100 µm as a stricter threshold, particularly for fixed restorations [6,7,8]. These criteria are typically applied to the region of interest relevant to prosthetic fit rather than entire arch reconstructions.

Although in vitro studies have established baseline accuracy for various IOS systems, they often fail to replicate real-world variables such as intraoral humidity, movement, and tissue complexity [9]. Thus, clinical evaluation of scanner performance is essential, particularly when scanning partially edentulous areas or when both teeth and mucosa are involved [10,11].

The accuracy of intraoral scanners may vary depending on the clinical indication and the extent of the scanned area. Previous studies have demonstrated that single-tooth and short-span digital impressions generally show higher accuracy compared with complete-arch scans due to reduced cumulative stitching errors and shorter scanning paths [3,9]. Conversely, complete-arch scans and partially edentulous situations tend to present greater challenges because of increased scan length and fewer stable anatomical landmarks, particularly in posterior regions [12]. Therefore, scanner performance should be evaluated within specific clinical scenarios to better understand its applicability in prosthodontic workflows.

This clinical study aimed to evaluate and compare the accuracy (trueness and precision) and scanning speed of three intraoral scanners—Medit i700, Primescan 1, and COXO DL-300P—in a controlled clinical setting. Two anatomically distinct scenarios were analyzed: the upper jaw, including prepared abutments and adjacent mucosa; and the lower jaw, limited to the abutment surfaces. The purpose was to determine whether scanners with varying costs can provide clinically acceptable outcomes in prosthodontic applications. Accordingly, this study aimed to compare the trueness, precision, and scanning speed of three intraoral scanners under standardized clinical conditions. Three null hypotheses were formulated: (1) no significant difference would exist among the scanners in trueness; (2) no significant difference would exist among the scanners in precision; and (3) no significant difference would exist among the scanners in scanning time.

## 2. Materials and Methods

This clinical study was conducted in accordance with the Declaration of Helsinki and with the ethical approval obtained from the Research Ethics Committee of the Victor Babes University of Medicine and Pharmacy Timisoara (Approval No.62/6 November 2025). The patient provided written informed consent for participation and for the use of clinical data and photo-documentation.

A 50-year-old male patient presented for fixed prosthetic rehabilitation of the missing maxillary left first molar and premolar (teeth 2.6 and 2.4), and mandibular left first and second molars (3.6 and 3.7). The patient declined implant therapy and opted for fixed prostheses on natural abutments. Vertical preparations were performed on abutment teeth 2.5 and 2.7 (upper jaw), and 3.5 and 3.8 (lower jaw).

Three intraoral scanners were used in this study: COXO DL-300P (COXO Medical Instrument Co., Ltd., FOSHAN, Guangdong, China), Medit i700 (Medit Corp., Seoul, South Korea), and Primescan 1 (Dentsply Sirona, Bensheim, Germany). Prior to initiating the scanning procedures, each scanner was calibrated according to the manufacturer’s specifications to ensure optimal performance and consistency across all scans.

The main characteristics of the scanners and software used in this study, including their reported optical acquisition technologies, are summarized in Table 1.

A total of 10 complete scans were performed per scanner, executed in a rotational sequence (Coxo → Medit → Primescan), maintaining identical environmental conditions throughout. All scans were conducted on the same patient during a single clinical session, with the same experienced prosthodontist performing all digital impressions to eliminate operator-related variability. The clinical environment was standardized to minimize external variability during digital impression acquisition. All scans were performed in the same treatment room using only ambient LED ceiling lighting, without activation of the dental unit operatory light. The patient remained in a stable semi-supine position throughout the entire scanning session, with no repositioning between scans. Moisture control was maintained using high-volume suction and compressed air, while cheek and lip retraction was achieved using an Optragate^®^ (Ivoclar Vivadent AG, Schaan, Liechtenstein) device to ensure consistent field visibility.

A consistent scanning strategy was maintained for all scanners and repetitions to minimize operator-related variability. For each arch, scanning began at the most posterior aspect of the prepared abutment region and progressed along the occlusal surfaces toward the anterior segments, followed by buccal and lingual sweeps to capture the remaining tooth and soft tissue surfaces until the entire prosthetic field had been captured in a single pass. The operator maintained a continuous scanning motion and a consistent scanner-to-surface distance as recommended by the manufacturers. No rescanning, trimming, or post-acquisition editing was allowed during the scanning procedure. This approach ensured consistency in evaluating scanning time and accuracy.

In addition to the arch scans, interocclusal registration (occlusion) was performed for each scan set using the scanner’s native bite registration protocol. Although not analyzed or timed in this study, occlusal data acquisition ensured completeness of the clinical digital workflow.

Scanning time was measured independently for each arch using a SEIKO S056 digital stopwatch (Seiko Holdings Corp., Tokyo, Japan). The timer was started when the first live scan data appeared on-screen and stopped when the scan acquisition process ended. Timing was recorded in seconds for each arch, providing a consistent metric of scanning efficiency across all scanner groups.

To establish a high-accuracy baseline for the evaluation of trueness, a conventional polyvinyl siloxane (PVS) impression was performed for both arches following the digital scanning procedures. A two-viscosity technique was applied using V-Posil Putty Fast and V-Posil Light Fast (VOCO GmbH, Cuxhaven, Germany). The impression was taken using a perforated full-arch stock tray under controlled conditions and in accordance with the manufacturer’s instructions for working and setting times, ensuring optimal dimensional stability and detail reproduction.

**Figure 1:**
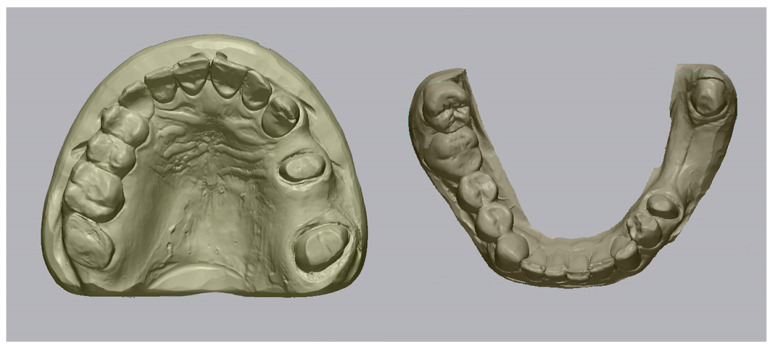
Digitized cast models designated as the reference datasets.

Following polymerization, the impression was poured with a type IV high-strength dental stone (Fujirock EP, GC Corporation, Tokyo, Japan) to fabricate a master cast. After a complete setting time of 60 min, the stone models were digitized using a high-precision desktop scanner (Medit T300, Medit Corp., Seoul, Republic of Korea), which provides an accuracy of less than 7 µm under ISO 12836 standards [13]. The resulting STL files of the digitized casts were designated as the reference datasets for all subsequent accuracy evaluations, representing a high-accuracy reference model (Figure 1).

To ensure meaningful and clinically relevant comparisons between the intraoral scans and the reference models, the analysis of trueness and precision was limited to well-defined areas of interest rather than full-arch datasets.

**Figure 2:**
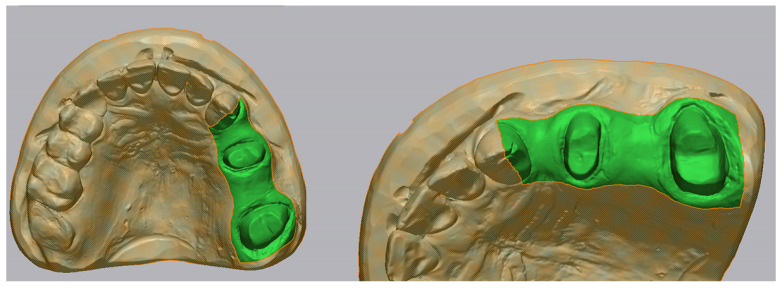
Isolated region of interest on the upper jaw.

For the upper jaw, the region of interest included the entire surface of the prepared abutment teeth 2.5 and 2.7, the distal half of the mesial neighboring tooth (2.3), and the surrounding soft tissue areas extending 5 mm apically along the buccal and palatal slopes (Figure 2). This anatomical configuration was chosen to reflect the clinical complexity of scanning both hard and soft tissues in partially edentulous maxillary regions, which are often prone to digital distortion.

**Figure 3:**
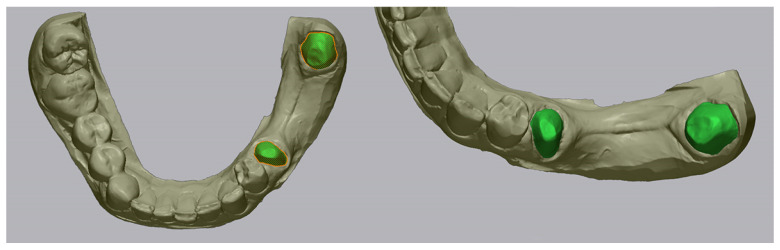
Isolated region of interest on the lower jaw.

For the lower jaw, the area of interest was limited exclusively to the prepared surfaces of abutment teeth 3.5 and 3.8, excluding adjacent teeth and mucosa (Figure 3). This isolated configuration was selected to evaluate scanner accuracy in a more controlled, hard-tissue-only environment, reducing variables introduced by soft tissue dynamics.

All isolations were performed using Geomagic Control X (Version: 16.0.2.16496, 3D Systems, Wilsonville, OR, USA). The software enabled precise mesh segmentation and consistent area application by allowing the fixed retention of the reference area settings. Once defined on the reference dataset, the same area of interest was automatically applied to each subsequent test mesh, ensuring uniformity in all comparisons. This consistency was critical for ensuring that deviations in measurements were attributable to the scanners rather than to variability in analyzed geometry.

**Figure 4:**
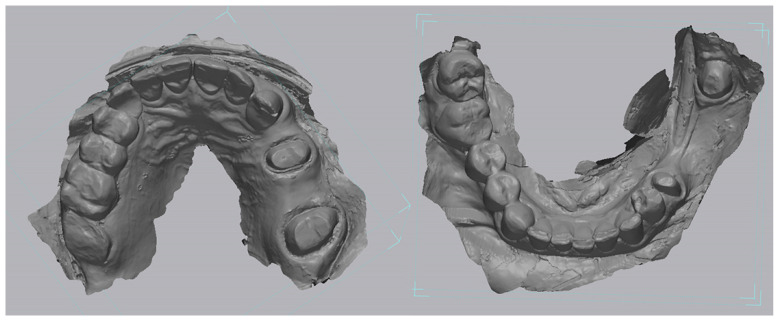
Primescan 1 intraoral scan file inside Geomagic Control X software.

**Figure 5:**
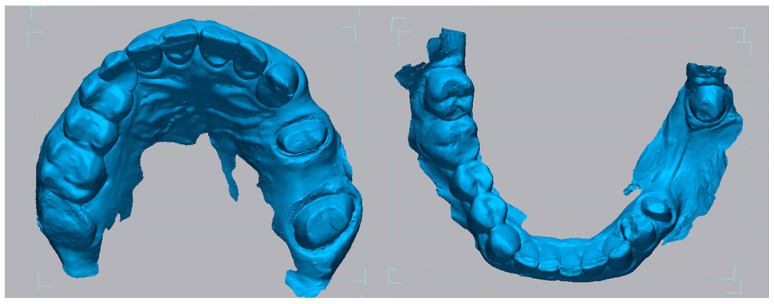
Medit i700 intraoral scan file inside Geomagic Control X software.

**Figure 6:**
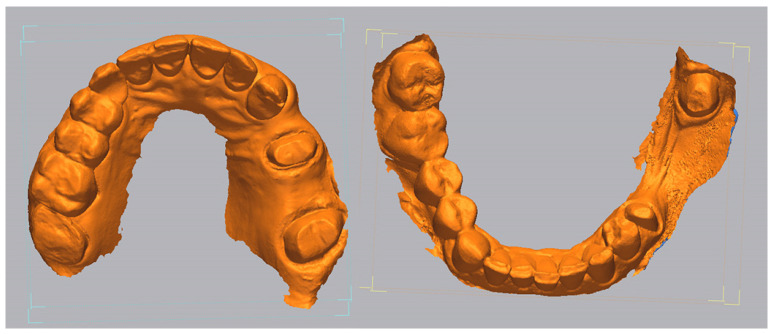
Coxo DL-300P intraoral scan file inside Geomagic Control X software.

All digital scan datasets (measured meshes) obtained from the three intraoral scanners were analyzed in comparison with the reference models using Geomagic Control X, a professional metrology and inspection software. The analysis was conducted separately for the upper and lower jaws, within the predefined areas of interest, as previously described. A visual example of the STL files, from each intraoral scanner, imported inside Geomagic Control X software, is presented in Figure 4, Figure 5 and Figure 6.

**Figure 7:**
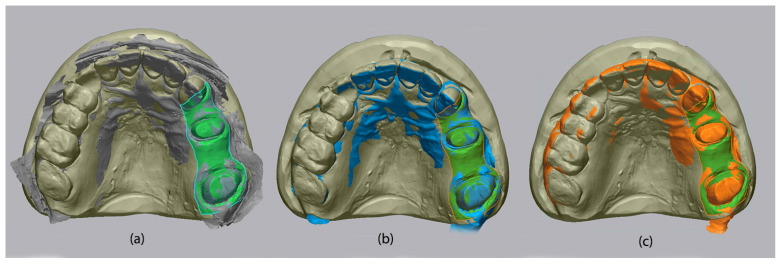
Superimposition of the measured meshes over the reference meshes, from left to right: Primescan 1 (**a**), Medit i700 (**b**), Coxo DL-300P (**c**).

Trueness was evaluated by superimposing each individual intraoral scan (*n* = 10 per scanner) over the corresponding reference model. Each comparison followed a two-step alignment procedure: an initial rough alignment, termed “Initial Alignment,” was followed by a refined “Best Fit Alignment,” which minimized the distance between point clouds within the defined region of interest (Figure 7). This process generated a high-precision overlay used for deviation analysis.

Precision was assessed by comparing all intraoral scans within each scanner group to each other, without reference to the analog model. These intra-group comparisons were conducted in the same manner as for trueness, using the best-fit alignment within the same area of interest for consistency.

**Figure 8:**
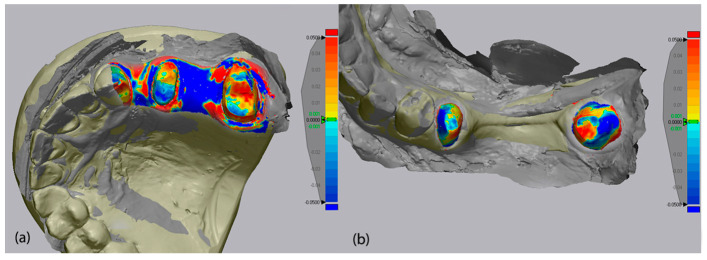
Color-coded deviation map of Primescan 1 for upper jaw (**a**) and lower jaw (**b**) analysis in parallel.

**Figure 9:**
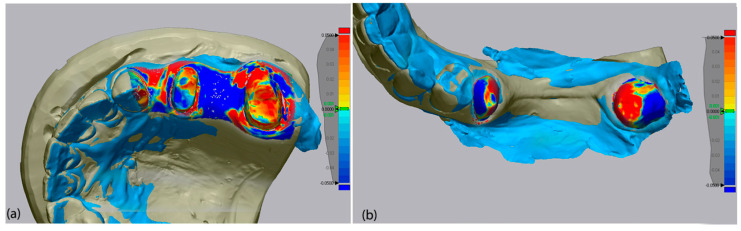
Color-coded deviation map of Medit i700 for upper jaw (**a**) and lower jaw (**b**) analysis in parallel.

**Figure 10:**
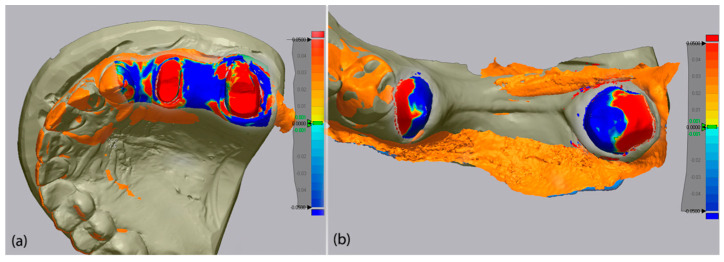
Color-coded deviation map of Coxo DL-300P for upper jaw (**a**) and lower jaw (**b**) analysis in parallel.

Following alignment, the software’s “3D Compare” function was employed to calculate Root Mean Square (RMS) deviation values, which quantify the spatial deviation between mesh surfaces. RMS deviation results were reported in millimeters (mm), and a ±0.05 mm (±50 µm) threshold was used for color-coded deviation maps to visually represent dimensional discrepancies. In these maps, green indicated minimal deviation (<±1 µm), blue represented negative (inward) deviation, and red indicated positive (outward) deviation beyond the threshold. Visual data of the color-coded deviation map of each scanner is presented in Figure 8, Figure 9 and Figure 10.

To ensure repeatability and comparability, all settings within the software were kept constant across all evaluations. Only the test meshes (measured data) were changed, while the reference mesh and area of interest remained fixed throughout each series of comparisons.

All trueness and precision values obtained from the 3D deviation analysis were exported into MedCalc Statistical Software (Version 20.218, MedCalc Software Ltd., Ostend, Belgium) for further statistical evaluation. Data were analyzed separately for the upper jaw (abutments and mucosa) and lower jaw (abutment surfaces only).

The Kolmogorov–Smirnov test was applied to assess the normality of data distributions for each dataset. Based on this assessment, the trueness values for both arches were found to follow a normal distribution, while the precision values exhibited non-normal distributions.

Accordingly, the trueness values were compared across the three intraoral scanner groups using One-Way Analysis of Variance (ANOVA). Where significant differences were detected, Student–Newman–Keuls post hoc tests were applied to determine intergroup significance.

For precision values, which did not meet the assumptions for parametric testing, a Kruskal–Wallis test was used as a non-parametric alternative. Post hoc comparisons were conducted using pairwise rank analysis as implemented by MedCalc, highlighting statistically significant differences between scanner groups.

The scanning time data were subjected to the same normality evaluation and were analyzed using One-Way ANOVA, given their parametric distribution. A significance level of α = 0.05 was adopted for all tests.

## 3. Results

The results of the Upper Jaw analysis are presented in Table 2.

**Figure 11:**
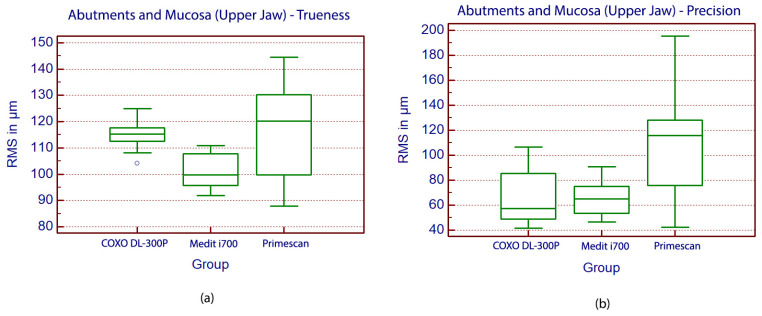
Boxplot displaying the values of trueness (**a**) and precision (**b**) in the Upper Jaw analysis. The small open circle represents an outlier, defined as an observation lying beyond 1.5 times the interquartile range from the quartiles. This value deviates substantially from the central distribution of the dataset. This outlier indicates a measurement that is unusually low compared to the rest of the COXO DL-300P group and may reflect variability, measurement error, or a true extreme value.

In the maxillary arch analysis, where the area of interest included the prepared abutments and adjacent mucosal regions, statistically significant differences in trueness were observed among the three intraoral scanners (*p* = 0.008, ANOVA). The Medit i700 demonstrated the highest trueness, with a mean deviation of 100.3 ± 6.6 µm, significantly outperforming both the Primescan 1 at 116.1 ± 17.7 µm, and the COXO DL- at 115.0 ± 5.8 µm, as determined by Student–Newman–Keuls post hoc analysis (Figure 11).

In evaluating precision within the maxillary arch, statistically significant differences were observed among the intraoral scanners (*p* < 0.0001, Kruskal–Wallis). Primescan demonstrated the highest variability, with a precision value of 115.7 µm, significantly less consistent than both the Medit i700 at 64.9 µm, and the COXO DL-300P at 57.4 µm (Figure 11).

The results of the Lower Jaw analysis are presented in Table 3.

**Figure 12:**
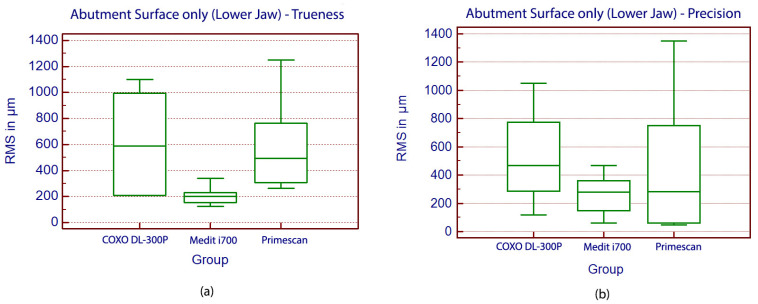
Boxplot displaying the values of trueness (**a**) and precision (**b**) in the Lower Jaw analysis.

For the mandibular arch, where the analysis focused exclusively on the abutment surfaces, significant differences in trueness were observed among the three scanners (*p* = 0.005, ANOVA). The Medit i700 demonstrated the best performance, with a mean trueness deviation of 193.1 ± 63.4 µm. Both Primescan 1 and COXO DL-300P exhibited substantially higher trueness deviations of 505.2 ± 291.5 µm and 492.8 ± 347.2 µm, respectively, as confirmed by Student–Newman–Keuls post hoc analysis (Figure 12).

The precision evaluation revealed significant differences among the scanners overall (*p* = 0.0004, Kruskal–Wallis with post hoc pairwise comparisons). Medit i700 achieved the greatest repeatability with a median deviation of 280.3 µm (IQR: 207.8 µm), followed closely by the Primescan 1 at 282.0 µm (IQR: 687.3 µm). The difference between Medit i700 and Primescan 1 was not statistically significant in this analysis, indicating comparable precision performance between these two scanners. In contrast, the COXO DL-300P showed significantly higher variability, with a median of 467.8 µm (IQR: 482.9 µm), underscoring its less consistent performance in this clinical setting (Figure 12).

The Scanning Time Results are presented in Table 4.

**Figure 13:**
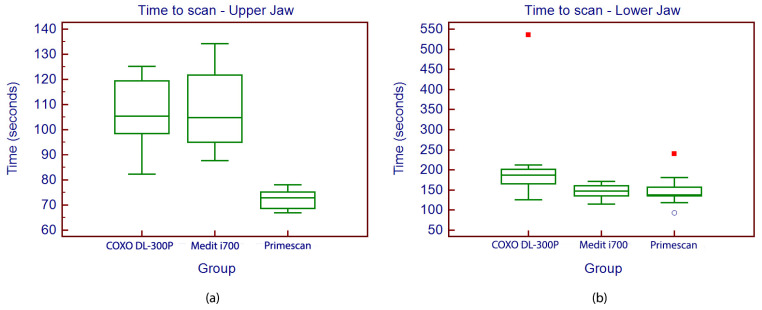
Boxplot displaying the values of scanning time of the upper jaw (**a**) and lower jaw (**b**). Outliers in the boxplots are displayed using two distinct symbols generated automatically by MedCalc statistical software. Both symbols represent observations that fall outside the typical distribution of the data based on the interquartile range (IQR) method. Mild outliers are indicated by open circles (○) and correspond to values lying between 1.5 and 3 times the IQR below the first quartile (Q1) or above the third quartile (Q3). Extreme outliers are indicated by filled red squares (■) and represent values that exceed 3 times the IQR from the quartiles. While both markings denote outlying observations, the red squares identify more extreme deviations from the central data distribution compared to the open circles. In this specific case the filled red squares denote extreme outliers (>!3×IQR), representing scan times substantially higher than the central distribution.

When evaluating the scanning time for the full-arch scan protocol, the results revealed differences among the three scanners for both arches. In the maxillary arch, Primescan 1 consistently demonstrated the fastest scanning times with an average scan time of 72.5 ± 3.8 s, being significantly faster than Medit i700 (107.2 ± 15.2 s) and COXO DL-300P (105.4 ± 13.5 s), as confirmed by the statistical analysis (*p* = 0.001) (Figure 13).

For the mandibular arch, differences between scanners did not reach statistical significance (*p* = 0.068). Primescan 1 achieved the shortest average time (144.3 ± 37.4 s), closely followed by an insignificant difference by Medit i700 (144.8 ± 17.2 s), while COXO DL-300P required 195.5 ± 109.9 s (Figure 13).

## 4. Discussion

This study compared the accuracy and scanning speed of three intraoral scanners under standardized clinical conditions. The findings showed that the Medit i700 achieved the highest trueness in both arches, Primescan delivered the fastest scans but with greater variability, and COXO demonstrated acceptable yet inconsistent precision. Based on the obtained results, the null hypotheses were partially rejected. The hypotheses stating that no significant differences would exist among the scanners in terms of trueness and precision were rejected, as statistically significant differences were observed between devices in both analyses. Regarding scanning time, the null hypothesis was rejected for the maxillary arch, where significant differences between scanners were detected, but it could not be rejected for the mandibular arch, where differences did not reach statistical significance.

The findings provide insights into how these devices perform in capturing digital impressions of both maxillary and mandibular arches, considering factors such as scanner generation, scanning protocols, and anatomical challenges.

### 4.1. Accuracy in Maxillary and Mandibular Scans

The Medit i700 demonstrated superior trueness in both arches, with values of 100.3 ± 6.6 µm for the maxilla and 193.1 ± 63.4 µm for the mandible. These results suggest that newer-generation scanners like the Medit i700 can achieve high trueness levels, potentially due to advancements in hardware and software integration. In contrast, the Primescan 1 and COXO DL-300P exhibited higher deviation values, particularly in the mandibular arch, where they recorded trueness values of 505.2 ± 291.5 µm and 492.8 ± 347.2 µm, respectively.

These findings align with previous research indicating that scanning larger areas, such as full arches, can introduce more errors compared to smaller scan regions like individual abutments. A study by Moon et al. (2020) found that complete-arch scans showed greater errors (0.09–0.10 mm) compared to quadrant scans (0.05–0.06 mm), with increased inaccuracies observed in posterior regions [12].

The magnitude of the trueness deviations observed in this study should also be interpreted in the context of clinically acceptable thresholds reported in prosthodontic literature. Marginal and internal fit values between approximately 50 and 120 µm are generally considered clinically acceptable for fixed restorations [6,8]. However, it is important to note that the deviation values reported in digital scan comparisons represent geometric discrepancies between digital datasets rather than the final marginal adaptation of a prosthetic restoration. Consequently, larger deviation values may be observed in full-arch digital impressions due to cumulative stitching errors and extended scanning paths, particularly in partially edentulous posterior regions [9].

### 4.2. Challenges in Scanning Edentulous and Posterior Regions

The abutment positions of the clinical case presented in the current study were selected because they created clinically relevant posterior scanning scenarios involving partially edentulous areas with both shorter and longer spans, allowing the evaluation of scanner performance in regions where intraoral scanning is typically more challenging due to increased scanning distance and reduced anatomical landmarks.

The inclusion of edentulous areas and posterior regions in the scan poses additional challenges for IOS devices. These areas often lack distinct anatomical landmarks, making it difficult for scanners to accurately capture the soft tissue morphology. Moreover, limited space in posterior regions can hinder the scanner’s ability to maintain optimal angulation and distance, potentially compromising data acquisition. These factors may contribute to the higher deviation values observed in the mandibular scans of the Primescan 1 and COXO DL-300P. Studies have shown that the accuracy of intraoral scanners can be influenced by various factors, including the presence of edentulous areas and the location within the oral cavity. For instance, a scoping review by Achmadi et al. highlighted that morphological differences in edentulous arches could impact scanning outcomes, with intraoral scanners demonstrating considerable promise but also facing challenges in certain conditions [14]. Similarly, a systematic review by Srivastava et al. found that the accuracy of intraoral scanners in recording completely edentulous arches varied, with peripheral borders and poorly traceable structures like the soft palate showing maximum discrepancies [15]. Furthermore, the accuracy of intraoral scanning is influenced by various factors such as scanner selection, operator skill, calibration, patient’s oral anatomy, ambient conditions, and scanning aids [16].

Repeated scanning protocols are commonly used in studies evaluating intraoral scanner accuracy to assess precision through the comparison of multiple acquisitions of the same object. Several investigations have used repeated scans per scanner, often around ten acquisitions, to allow reliable estimation of repeatability and deviation values [17,18,19].

### 4.3. Impact of Scanner Generations

The three scanners evaluated in this study represent different generations of IOS technology: Primescan 1 was released in 2019, Medit i700 in 2021, and COXO DL-300P in 2024. Advancements in scanner technology over time have led to improvements in accuracy, speed, and user experience. Recent studies have demonstrated that newer-generation IOS devices tend to exhibit improved accuracy and precision compared to their predecessors. For instance, a comparative analysis of four different intraoral scanners revealed that the Medit i700 and Trios 5 exhibited balanced performance with average deviations of 114 µm and 112 µm, respectively, while the Primescan displayed a higher deviation of 127 µm [20].

The Medit i700’s superior performance may be attributed to such technological enhancements, despite its lower cost compared to the Primescan 1. This observation underscores the importance of considering both the release date and technological capabilities of IOS devices when selecting equipment for clinical use. It is worth mentioning that Coxo DL-300P is the most recently released and has the lowest price. Although it failed to outperform the Medit i700, it performed close to Primescan 1’s level regarding trueness of both analyses. This could imply that new generations of intraoral scanners, even at a low production price, may offer clinically acceptable accuracy performance comparable to older generation scanners that have endured the test of time.

Differences in the optical acquisition technologies used by intraoral scanners may also influence scanning performance and accuracy outcomes. Modern intraoral scanners rely on various imaging principles such as confocal microscopy, triangulation, structured light projection, and high-frequency contrast analysis to reconstruct three-dimensional dental structures. These technologies differ in their ability to capture surface geometry, maintain scanner tracking, and manage reflective or moist intraoral environments, which may ultimately affect the trueness and precision of the resulting digital impressions. For example, confocal-based systems capture depth information by focusing at different distances, whereas structured light systems reconstruct geometry by projecting patterns onto the scanned surface and analyzing the resulting distortion. These technological differences may contribute to variations in scanning accuracy, particularly in extended scans or in anatomically complex regions such as posterior or partially edentulous areas [1,4]. However, it should be noted that all scanners evaluated in the present study belong to the structured-light/triangulation category of intraoral scanners. Therefore, the observed differences in performance are more likely related to proprietary implementation factors such as sensor configuration, camera arrangement, acquisition speed, and image-processing algorithms rather than fundamentally different optical scanning principles.

### 4.4. Calibration Protocols and Their Importance

Calibration plays a crucial role in maintaining the accuracy of IOS devices; therefore, regular calibration ensures that the scanner’s sensors provide accurate and consistent results, which is essential for producing reliable digital impressions [21]. Neglecting calibration can lead to significant reductions in scanning accuracy, potentially compromising the quality of dental restorations and overall patient care. Both the Primescan 1 and Medit i700 utilize dedicated calibration units to ensure consistent performance. In contrast, the COXO DL-300P is marketed as a self-calibrating device, lacking an external calibration unit. While self-calibration may offer convenience, it raises concerns about long-term accuracy maintenance, especially with frequent use. It is noteworthy that the COXO DL-300P used in this study was relatively new and had not undergone extensive use, suggesting that its performance reflects its initial calibration state. It is important to monitor through future studies how the new generations of scanners behave over time and if they will stand the test of time, especially if they can retain their accuracy even without dedicated calibration devices.

### 4.5. Considerations Regarding the Reference Model

This study employed a conventional analog impression technique using polyvinyl siloxane (PVS) material to create a reference model. PVS is renowned for its high-dimensional stability and accuracy, making it a gold standard in impression materials. Previous studies have demonstrated that polyvinyl siloxane impression materials exhibit excellent dimensional stability and remain among the most reliable materials for obtaining accurate dental casts [22]. Moreover, similar conventional impression–cast digitization protocols have been used as reference datasets in several investigations evaluating the accuracy of intraoral scanners [9]. The impressions were poured using type IV dental stone, known for its minimal expansion and high strength, and subsequently scanned with a high-precision desktop scanner [23]. While each step introduces potential sources of error, the materials and methods chosen are considered among the most accurate in clinical practice. Nonetheless, it is essential to acknowledge that any inaccuracies in the reference model could influence the assessment of IOS performance.

### 4.6. Scanning Time and Clinical Efficiency

Efficiency in clinical workflows is paramount, particularly when integrating digital technologies such as intraoral scanners (IOS). In this study, Primescan 1 exhibited the shortest scanning times for the maxillary arch, completing scans significantly faster than the Medit i700 and COXO DL-300P. However, the differences in scanning time among the scanners were not statistically significant for the mandibular arch, reflecting the particular challenges associated with that region.

The longer scanning times observed in the mandibular arch were likely influenced by the presence of a large edentulous area extending from the 3.5 to 3.8 region. This anatomical scenario consistently presented difficulties for all three scanners tested, as they frequently lost track of the scanning area, requiring repeated adjustments and rescans. Such challenges are well-documented in the literature, with studies indicating that edentulous regions, particularly in the posterior areas, pose significant obstacles for IOS devices due to the lack of distinct anatomical landmarks and limited space for maneuvering the scanner tip [24].

While Primescan 1 achieved efficient scanning in areas with sufficient abutments and only short edentulous spans, it struggled, just like the Medit i700 and COXO DL-300P, in capturing accurate data when faced with a large edentulous region. These observations suggest that even advanced intraoral scanners experience diminished efficiency and reliability when encountering extensive gaps without clear anatomical references. This underscores the importance of careful scanning strategies and patient-specific considerations in clinical practice; therefore, clinicians should be aware of these limitations and consider incorporating supplementary techniques or tools to enhance scanning accuracy and efficiency in challenging anatomical scenarios.

### 4.7. Study Limitations and Future Directions

The findings of the present study should be interpreted within several limitations. First, the investigation was conducted on a single patient, which limits the generalizability of the results to broader clinical populations. Second, only one specific prosthodontic scenario involving posterior abutments and partially edentulous areas was evaluated. Third, all scans were obtained during a single uninterrupted clinical session to maintain identical environmental conditions across acquisitions. Additionally, this design may have introduced potential influences such as patient fatigue, operator fatigue, or progressive difficulty in maintaining optimal isolation of the working field.

Finally, the reference dataset was generated using a conventional impression and stone cast, subsequently digitized with a high-accuracy desktop scanner, which represents a reliable clinical reference but may still introduce minor cumulative deviations. Future studies including larger patient samples, multiple operators, and different clinical scenarios are required to further validate these findings.

The results of this study highlight that intraoral scanner performance may vary depending on both device implementation and clinical conditions such as scan length and anatomical complexity. In prosthodontic workflows involving prepared abutments and partially edentulous regions, clinicians should consider not only scanning speed but also the consistency and trueness of digital impressions. Understanding these performance characteristics may assist clinicians in selecting appropriate scanning systems and strategies to improve the reliability of digital impressions used for fixed prosthetic rehabilitation.

## 5. Conclusions

Within the limitations of this preliminary single-patient clinical study, the following conclusions can be drawn:

1. The Medit i700 consistently demonstrated the highest trueness and precision across both the maxillary and mandibular arches, outperforming the Primescan 1 and COXO DL-300P.
2. Primescan 1 exhibited the fastest scanning times for the maxillary arch, although these differences were not statistically significant for the mandibular arch.
3. All three intraoral scanners faced notable challenges in scanning large edentulous areas, especially in the posterior mandible, resulting in increased scan times and decreased accuracy.

These conclusions highlight the importance of considering both the technical capabilities of scanners and specific clinical scenarios when selecting and using IOS devices in practice.

## Figures and Tables

**Table 1:** Devices and software used in the study.

Device/Software	Manufacturer	Type/Role in Study	Optical Technology/Principle
Primescan 1	Dentsply Sirona (Germany)	Intraoral scanner	Structured light + triangulation (high-frequency dynamic depth scanning with Smart Pixel sensor)
Medit i700	Medit Corp. (South Korea)	Intraoral scanner	Structured light + triangulation (dual-camera structured-light projection system—Medit optical engine)
Coxo DL-300P	COXO Medical Instrument Co. (China)	Intraoral scanner	Structured light + triangulation (LED structured-light projection with video capture reconstruction)
Medit T300	Medit Corp. (Republic of Korea)	Desktop scanner (reference model digitization)	Structured light + triangulation (multi-camera structured-light scanning with rotating stage for model capture)
Geomagic Control X	3D Systems (USA)	3D metrology software	Mesh comparison and deviation analysis
MedCalc Statistical Software	MedCalc Software Ltd. (Belgium)	Statistical analysis	Statistical analysis software

**Table 2:** Result of Upper Jaw (Abutments and Mucosa) analysis in micrometers (µm).

Upper Jaw (Abutments and Mucosa)	Trueness		Precision	
	Mean	SD	Median	IQR
PRIMESCAN 1	116.1 µm	17.7 µm	115.7 µm	51.6 µm
MEDIT i700	100.3 µm	6.6 µm	64.9 µm	21.5 µm
COXO DL-300P	115.0 µm	5.8 µm	57.4 µm	36.3 µm

**Table 3:** Results of Lower jaw (Abutment Surface only) analysis in micrometers (µm).

Lower Jaw (Abutment Surface Only)	Trueness		Precision	
	Mean	SD	Median	IQR
COXO DL-300P	492.8 µm	347.2 µm	467.8 µm	482.9 µm
MEDIT i700	193.1 µm	63.4 µm	280.3 µm	207.8 µm
PRIMESCAN 1	505.2 µm	291.5 µm	282.0 µm	687.3 µm

**Table 4:** Scanning Time Results of full-arch scanning protocol measured in seconds (s).

Full-Arch Scanning Time	Upper Jaw		Lower Jaw	
	Mean	SD	Mean	SD
COXO DL-300P	105.4 s	13.5 s	197.5 s	109.9 s
MEDIT i700	107.2 s	15.2 s	144.8 s	17.2 s
PRIMESCAN 1	72.5 s	3.8 s	144.3 s	37.4 s

## Data Availability

The data presented in this study are available on request from the corresponding author. The data are not publicly available due to ethical restrictions and patient privacy considerations, as they contain clinical data and intraoral scan datasets derived from a patient involved in this study.
